# Brain connectivity changes occurring following cognitive behavioural therapy for psychosis predict long-term recovery

**DOI:** 10.1038/tp.2016.263

**Published:** 2017-01-17

**Authors:** L Mason, E Peters, S C Williams, V Kumari

**Affiliations:** 1 Department of Psychology, Institute of Psychiatry, Psychology and Neuroscience, King’s College London, London, UK; 2 South London and Maudsley NHS Foundation Trust, NIHR Biomedical Research Centre for Mental Health, London, UK; 3 Department of Neuroimaging, Institute of Psychiatry, Psychology and Neuroscience, King’s College London, London, UK

## Abstract

Little is known about the psychobiological mechanisms of cognitive behavioural therapy for psychosis (CBTp) and which specific processes are key in predicting favourable long-term outcomes. Following theoretical models of psychosis, this proof-of-concept study investigated whether the long-term recovery path of CBTp completers can be predicted by the neural changes in threat-based social affective processing that occur during CBTp. We followed up 22 participants who had undergone a social affective processing task during functional magnetic resonance imaging along with self-report and clinician-administered symptom measures, before and after receiving CBTp. Monthly ratings of psychotic and affective symptoms were obtained retrospectively across 8 years since receiving CBTp, plus self-reported recovery at final follow-up. We investigated whether these long-term outcomes were predicted by CBTp-led changes in functional connections with dorsal prefrontal cortical and amygdala during the processing of threatening and prosocial facial affect. Although long-term psychotic symptoms were predicted by changes in prefrontal connections during prosocial facial affective processing, long-term affective symptoms were predicted by threat-related amygdalo-inferior parietal lobule connectivity. Greater increases in dorsolateral prefrontal cortex connectivity with amygdala following CBTp also predicted higher subjective ratings of recovery at long-term follow-up. These findings show that reorganisation occurring at the neural level following psychological therapy can predict the subsequent recovery path of people with psychosis across 8 years. This novel methodology shows promise for further studies with larger sample size, which are needed to better examine the sensitivity of psychobiological processes, in comparison to existing clinical measures, in predicting long-term outcomes.

## Introduction

Psychotic experiences can be highly distressing and people experiencing psychosis often also show high levels of emotional disturbances.^1^ Although effective pharmacological and psychological interventions exist, high rates of relapse remain,^2^ and residual symptoms and distress typically persevere between episodes, for example.^3^ Identifying the treatment factors that predict favourable recovery pathways is an important step towards improving future interventions.

An important step forward in evidence-based practice, across psychiatric disorders, has been the use of objective clinical measures for the purpose of outcome monitoring in individuals, therapists and services.^4^ Although increasingly important in service-level clinical decision-making, including allocation of resources and funding,^5^ these measures remain poor in predicting long-term outcomes. In psychosis, for example, a recent meta-analysis showed that both clinical and demographic variables are poor predictors of relapse, with non-significant effects observed for psychosis symptoms (either positive or negative), affective symptoms or clinician-rated insight.^6^ Measuring change at the level of the psychological processes that generate and maintain these symptoms may be helpful in improving the treatment evaluation as well as for predicting long-term outcomes.

Theoretical models of psychosis postulate that aberrant threat processing is key in generating and maintaining positive symptoms.^7, 8^ The psychological processes involved in threat are problematic to quantify by self-report measures due to subjective bias (both for patient and clinician). Functional neuroimaging has yielded robust and objective markers of threat processing in psychosis.^9, 10^ Recently, there has been increasing interest in utilising these psychobiological markers to investigate the neural mechanisms of psychological therapies (for reviews see Barsaglini *et al.*^11^and Mason *et al.*^12^). In psychosis, two reports have arisen from an investigation of cognitive behavioural therapy for psychosis (CBTp) compared with treatment-as-usual.^13, 14^ In the first study, we reported reduction in brain response to social threat from pre- to post- CBTp functional MRI (fMRI) measurements.^13^ Recently, we further showed that these activation changes were accompanied by reorganisation of numerous connections with prefrontal cortical and with several limbic brain regions.^14^ In line with our hypotheses, we found that connectivity between dorsolateral prefrontal cortex (DLPFC) and amygdala increased following CBTp. Under cognitive neuroscience models of emotion regulation, this could indicate an increased ability to contextualise potential social threat and thereby cognitively regulate negative affect,^15, 16^ which fits with psychological treatment models of CBTp.^10, 11^ An important finding was that the vast majority of connectivity changes did not correlate with symptom change, suggesting that they captured other CBTp-specific changes involved in socio-affective processing, over and above the symptom improvement captured by routine clinical measures. The two connectivity changes to correlate with improvement in psychotic symptoms were increases in DLPFC connectivity with inferior parietal lobule (IPL, when processing social threat) and with postcentral gyrus (when processing prosocial facial affect). The IPL has previously been associated with theory of mind and cognitive insight in schizophrenia,^14, 17, 18^ and cognitive insight, which includes self-reflectiveness, has been shown to increase following CBTp,^19^ providing a plausible route by which changes in functional threat-related connectivity may mediate improvement in positive psychotic symptoms. An unexpected finding was that symptom improvement was also associated with DLPFC-postcentral gyrus connectivity, which may be understood in terms of its putative involvement in the mirror neuron system, specifically in somatic aspects of empathy during the processing of facial affect.^14, 20^ In support of this, abnormalities in this region have been associated with deficits in emotion recognition^21, 22^ and to correlate with psychotic symptoms.^23^ In line with this view, we found the symptom association was present for the processing of prosocial (rather than threatening) facial affect, which may be related to the assertion that paranoia may be secondary to the misperception of benign affect as threatening, for example.^24^

In the present study, we sought to examine whether these CBTp-led changes in socio-affective processes are determinants of long-term clinical outcomes. To this end, we employed novel investigation methods to retrospectively follow-up a previously reported cohort^13, 14^ over ~8 years since they received CBTp. Given the high variability in psychotic symptoms over time, both in terms of relapse as well as between-episode fluctuation of residual symptoms,^3^ we obtained monthly measurements instead of relying on a single follow-up ‘snapshot’ (see ‘Materials and Methods’ section).

We predicted that the degree to which threat-related DLPFC-amygdala connectivity increased following CBTp would predict greater long-term remission in both positive psychotic symptoms and affective symptoms, given the importance of this connection in contextualising potential social threat and in regulating affect.^15, 16^ Being key to affective well-being, we further predicted that this connectivity would determine subjective ratings of recovery at long-term follow-up. Finally, we predicted that the CBTp-led increases in amygdalo-IPL and DLPFC-postcentral gyrus connectivity that had previously been associated with improvement in psychotic symptoms following CBTp,^14^ would predict greater levels of remission in this symptom domain.

## Materials and Methods

### Participants and design

Participants were 22 outpatients with a confirmed diagnosis of paranoid schizophrenia (final *N*=15; see Table 1) who had taken part in our earlier studies.^13, 14^ These participants had completed an fMRI implicit facial affective processing task and a battery of clinical measures on two occasions, pre (T1) and post (T2) receiving 6 months of CBTp. Sixteen outpatients receiving treatment-as-usual were also scanned at these time points (data not analysed as part of the present study).

### Procedure

We retrospectively followed up these participants as their final fMRI scan (at T2), an average of 8 years (range 7–9 years) before the current study (T3). We obtained objective clinical outcomes for this entire period through case note review (T2 to T3; see Longitudinal Clinician Ratings) as well as current subjective ratings of recovery and well-being (at T3; see Outcome Measures).

Consent was obtained by seeking current contact details from consultant clinicians in the services providing care for the previously recruited participants. Participants were then contacted by phone and those expressing an interest received information about the study, a consent form for accessing their electronic clinic records, self-report questionnaires assessing well-being and recovery and a prepaid envelope. The final sample of participants who consented and returned the questionnaires were reimbursed £10 for their time. Ethical approval was granted by the National Health Service research ethics committee (reference: 14/LO/0325).

#### Functional neuroimaging procedure

As described in earlier reports,^14^ participants were presented with monochrome faces depicting fear, anger, happiness or neutral expressions,^27^ and had to indicate the sex of the face with a button press response. These were repeated in four blocks per condition, with counterbalancing across 16 blocks (see Mason *et al.*^14^ and Supplementary Material for further details of the scanning protocol). Changes in functional connectivity from T1 to T2 were quantified during social threat (angry faces) and prosocial social affect (happy faces) using the psychophysiological interaction approach.^28^ Seeds were left amygdala and right DLPFC; whereas bilateral activation was found, we selected the regions of maximum task activation that were reported previously.^16^ Seeds were defined functionally from the group-level maxima, with spheres around these maxima (3 and 4 mm radius for amygdala and DLPFC, respectively). These were additionally constrained within anatomical masks for these regions as defined by the PickAtlas toolbox.^29^ Significant connectivity changes following CBTp were tested by examining the interaction of group (CBTp vs treatment-as-usual) by time (T1 vs T2). There were exclusively increases in connectivity in the CBTp group across functional connections with amygdala and DLPFC.

For the present study, we focused our analyses on the change in DLPFC-amygdala connectivity that occurred during social threat processing, because of the strong theoretical link with cognitive regulation of affect^15, 16^ and in turn the relevance to cognitive behavioural models of positive symptoms of psychosis.^7, 8^ We also included the two connectivity changes that previously correlated with improvement in positive psychotic symptoms: amygdala-IPL and DLPFC-postcentral gyrus, which had occurred for the processing of threat and prosocial facial affect, respectively.^14^

#### Cross-sectional clinical measures

The following clinician-administered and self-report measures had previously been administered pre- and post- CBTp (T1 and T2). The Positive and Negative Syndrome Schedule (PANSS);^26^ is a clinician-administered rating of positive, negative and general psychopathology symptoms. Affective symptoms were measured from the Beck Depression Inventory, second edition.^30^

We acquired additional measures at long-term follow-up (T3). We assessed subjective recovery using the Questionnaire about the Process of Recovery (QPR,^31^), a service-user led instrument that follows theoretical models of recovery and provides a measure of constructs such as hope, empowerment, confidence, connectedness to others. This was our primary measure as it has one of the best psychometric properties of recovery measures^32^ and can be expected to be relatively robust to fluctuations in clinical state, making it well suited to use a cross-sectional measurement of long-term outcome. Additional measures for well-being, satisfaction and functioning were acquired (Supplementary Materials) but were not included in analyses because of missing observations, a high correlation with self-reported recovery and to reduce the number of analyses reported. These data are available on request from the first author.

Longitudinal clinician ratings of symptoms: We retrospectively determined symptoms and functioning from electronic case note data held by local National Health Service trusts in South London. This covered the entire period between participants’ final fMRI measurements (T2; circa 2007) and January 2015 (T3). Two raters followed validated operationalised criteria^33^ to infer presence of positive psychotic symptoms for each month independently, based on clinical note entries made by mental health professionals. Participants were rated as being in ‘full remission’ (no symptoms present), ‘partial remission’ (symptoms of low intensity or frequency with clinicians noting at least partial insight), or ‘no remission’ (moderate symptoms; see Bebbington *et al.*^33^ for fully detailed criteria).

Ratings of affective symptoms were based on both the intensity and frequency of affective disturbance as follows. Affective symptoms were rated as ‘low’ when there was no indication of distress or only brief periods (<3 days, maximum of two separate instances for that month) of mild-to-moderate severity (without expression of suicidality and that did not require intervention by mental health professionals). ‘Moderate’ affective symptoms was rated where there was any period of distress lasting more than 3 days, where there was expression of suicidality not requiring severe management, or where there were three or more instances of ‘low’ affective symptoms present for that month. ‘Severe’ was rated for any month in which there was severe distress and suicidality requiring severe management, including hospitalisation or home treatment care. This method was shown to have high reliability and clinical validity,^33^ with strong associations between the ratings of symptoms made by case note ratings and PANSS in the same participants. We confirmed that reliability was also high for ratings made in the present study, with inter-rater agreement ranging from ‘moderate’ to ‘almost perfect’ (Supplementary Materials).

In addition to these symptom ratings, we also rated level of care needed (categories: care of general practitioner only; outpatient appointments in secondary care; daily home treatment; hospital treatment) and occupational functioning (paid employment; voluntary work or training course; unemployed), as a mean of validating the clinical ratings (Supplementary Table 1). There were significant positive associations between the non-remission measure and amount of severe care (hospitalisation and home treatment; see Supplementary Table 2).

### Data analysis

#### Prediction of long-term outcomes from functional connectivity changes

Multivariate analysis of variance (MANOVA, Wilk’s Lambda) was used to relate the longitudinal, month-by-month clinician ratings of psychotic and affective symptoms (T2 to T3) as well as subjective recovery (T3), to the functional connectivity changes (T1 to T2) as follows. All tests were performed one-tailed.

Percentage of months spent in each of the three symptom states was computed for positive psychotic symptoms (full, partial or non-remission) and for affective symptoms (low, moderate or severe). To simplify the analyses and to reduce model over-fitting, we computed a single residualised variable for each symptom domain (see Supplementary Materials for details). The effect of psychotic and affective symptom domains was tested separately, by entering the respective symptom variable as a regressor within MANOVA, along with our hypothesised changes in connectivity as dependent variables. Bonferroni correction was applied for multiple tests (p/2) across the two symptom domains and significant effects were followed up using correlation tests (Spearman; *r*_ρ_) to clarify the direction of associations.

We also performed an exploratory analysis to address the hypothesis that the therapeutic effects of CBTp would be better captured by changes to core threat processes than by short-term symptom reduction.^14, 34^ Because of the exploratory nature, this analysis is reported as Supplementary Material.

Finally, we separately tested the relationship between the functional connectivity changes and long-term subjective recovery, the total score of which was entered as a regressor into MANOVA with the functional connectivity changes as dependent variables.

## Results

### Long-term clinical outcomes

At long-term follow-up, consent was obtained to access case note data for 15 of the 22 CBTp group (age=37.9, s.d.=7.56; 11 male). This subsample did not differ in terms of response to CBT, either in terms of improvement in psychotic symptoms or in depressive symptoms, and additionally did not differ in task performance (Table 1). These participants evidenced high rates of remission, with an average of 93.5% of months spent in either full or partial remission and evidenced by low rates of affective symptoms overall, with an average of 88.2% of months with low affective symptoms (Supplementary Table 1). As expected, symptom remission was highly associated with level of care (Supplementary Table 2), with months in non-remission positively correlating with months receiving hospital care and months receiving intensive home treatment. Months in non-remission were also positively correlated with months of severe affective symptoms (Supplementary Table 2).

### Prediction of long-term outcomes from functional connectivity changes

#### Longitudinal positive psychotic symptoms

**Figure 1:**
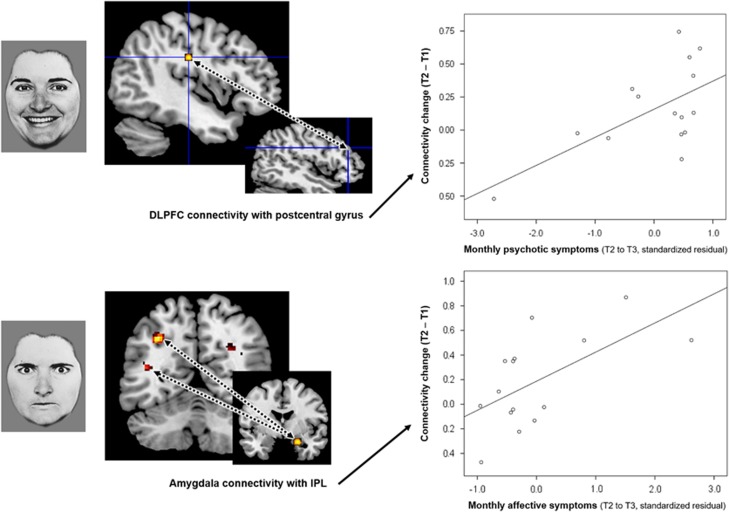
Following cognitive behavioural therapy for psychosis, the change in specific social-affect functional connections differentially predicts level of positive psychotic symptoms (top) and affective symptoms (bottom) across eight years. Top: the increase in connectivity between dorsolateral prefrontal cortex (DLPFC) and postcentral gyrus when processing prosocial facial affect predicted reduced levels of positive psychotic symptoms (*r*_ρ_(15)=0.495, *P*=0.06). Bottom: conversely, the increase in amygdala connectivity with the inferior parietal lobule (IPL) when processing social threat predicted reduced levels of affective symptoms (*r*_ρ_(15)=0.49, *P*=0.06). Dotted lines between brain regions represent connectivity.

Neither of the threat-related connections were significant in the model for long-term positive psychotic symptoms (*P*⩾0.42). There was a significant effect for the prosocial facial affect connection (change in DLPFC-postcentral gyrus connectivity; F(1, 13)=7.83, corrected-*P*=0.03), which was driven by a positive association (*r*_ρ_(15)=0.495, *P*=0.06; Figure 1). There was no multivariate level effect (corrected-*P*=0.12).

#### Longitudinal affective symptoms

There was a significant effect specifically for the change in threat-related connectivity between amygdala and IPL (F(1, 13)=7.72, corrected-*P*=0.032), but not DLPFC-amygdala connectivity (*P*>0.99). This significant effect was confirmed to be a positive association (*r*_ρ_(15)=0.49, *P*=0.06) (Figure 1). There was no effect for the change in prosocial facial affect connectivity (corrected*-P*=0.24). The multivariate level effect approached significance (F(3, 11)=4.35, corrected*-P*=0.06).

### Subjective long-term recovery

**Figure 2:**
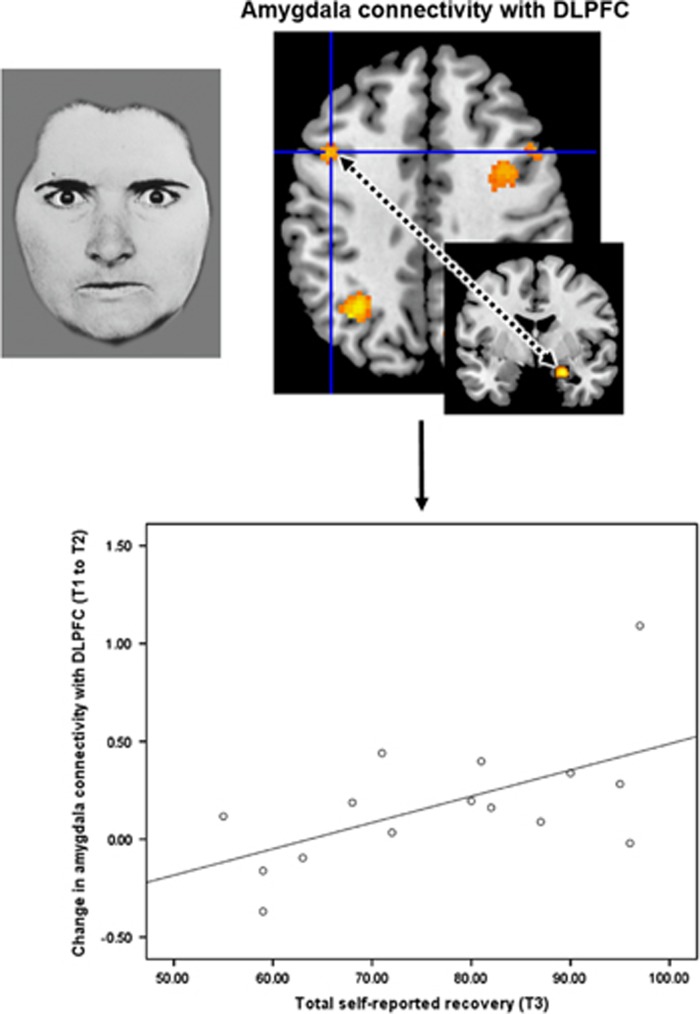
Functional connectivity changes following cognitive behavioural therapy for psychosis predict subjective recovery at 8-year follow-up. A greater increase in connectivity between amygdala and dorsolateral prefrontal cortex (DLPFC), when processing social threatening facial affect, was associated with higher levels of subjective recovery (*r*_ρ_(15)=0.51, *P*=0.05).

There was a significant effect specifically for the change in threat-related connectivity between amygdala and DLPFC (F(1, 13)=6.54, corrected-*P*=0.04), which was positively associated with long-term recovery (*r*_ρ_(15)=0.51, *P*=0.05) (Figure 2). There was no difference in the strength of association between the connectivity change and the ‘intrapersonal’ and ‘interpersonal’ subscales of recovery (*P*=0.82). There was no effect for the other connectivity changes (corrected-*P*⩾0.32) or at the multivariate level (corrected-*P*=0.11).

## Discussion

This study utilised an innovative methodology that combined functional neuroimaging with monthly clinician ratings of symptoms over a substantial eight-year period. We showed that the reorganisation that occurs at the neural level following psychological therapy can predict the subsequent recovery path of people with psychosis across this entire period (Figure 1).

In this study, the sole predictor of long-term positive psychotic symptoms was the degree to which prefrontal cortical connectivity with postcentral gyrus had been promoted following CBTp, specifically for the processing of prosocial (rather than threatening) facial affect (Figure 1, upper panel). When processing facial affect, this connection may integrate somatic aspects of affective empathy with higher-order appraisals.^14, 20^ It has been proposed that paranoia is causally linked to a tendency to misperceive benign affect as threatening, for example.^24^ Our finding that remission of positive psychotic symptoms (including paranoia) is determined by improvement in neural processes supporting affect recognition and empathy represents a novel psychobiological mechanism for CBTp.

Long-term affective symptoms were, on the other hand, predicted by a separate connection involved in the processing of potential social threat, specifically the degree to which connections had strengthened between amygdala and IPL (Figure 1, lower panel). Functional IPL networks have been linked to the allocation of attention^35^ as well as to theory of mind,^36^ and so one interpretation of the present findings would be that the ability to adequately allocate attention to and engage with the affect of others is important for long-term emotional well-being. Our hypothesis that affective symptoms would be predicted by top-down cognitive regulation of affective regions, putatively instantiated in DLPFC-amygdala connectivity, was not supported however. This connection did, however, predict participants’ subjective sense of recovery at long-term follow-up (Figure 2). It seems plausible to conclude that being better able to cognitively regulate negative emotion, especially in response to potential threat, is an important CBTp outcome that determines personally perceived recovery in the long run. This is concordant with service user-focused research, which has highlighted that the ability to better manage negative emotionality is an important feature of recovery for people with psychosis.^31^ Overall, these findings highlight that neural changes following CBTp confer a long-lasting benefit.

The possibility that separate psychobiological mechanisms mediate long-term affective and psychotic symptom domains builds on the view that CBTp can effectively alleviate distress and affective disturbances without necessarily altering psychotic symptoms themselves, for example^37^ and raises the possibility that changes in threat-related processing, specifically amygdalo-IPL connectivity, may be sufficient for long-term emotional well-being. This interpretation should be treated as preliminary until replicated because participants here had evidenced improvements in psychotic symptoms following CBTp (and not just affective symptoms; Table 1) and our supplementary analysis found evidence of common connections predicting both symptom domains (Supplementary Materials).

One of our aims was to establish to incremental value of using change in psychobiological processes as predictors of long-term outcomes over existing clinical measures. Although the final sample size was relatively small, and so replication is needed, we found preliminary evidence that these changes in socio-affective processing may be superior to short-term improvements in symptoms in predicting people’s long-term symptoms and recovery profiles (Supplementary Materials). Measuring change at the level of the psychological processes that are theorised to generate and maintain symptoms may be a more informative means of evaluating treatment.

We believe this is the first investigation of its kind in psychosis and also extends two longitudinal studies in anxiety disorders, which used a single follow-up time point, 6–12 months after psychological therapy.^38, 39^ Consistent with the CBTp-led promotion of a putatively amygdala-modulating circuit reported here, Furmark *et al.*^39^ found that CBT-led reductions in amygdalo-limbic activation predicted better clinical outcomes in social anxiety disorder patients. The same group have also shown limbic connectivity to be important in predicting treatment response, using a similar socio-affective processing task to ours.^40^ This overlap in psychobiological processes is consistent with the view that anxiety is inherent in the formation and maintenance of paranoia.^41^

There are several limitations to note of the present study. First, as is typical for follow-up studies, especially of this timespan, there was a high attrition rate. Although the final sample size remained adequate according to recommendations, for example,^42^ further work with greater statistical power will be needed to replicate and extend the present findings, in particular to explore how the connectivity changes differentially associate with symptom domains. Second, although the facial affective processing task is widely utilised in clinical research to elicit threat processing, future work with more nuanced designs will be necessary to further elucidate the functional significance of these brain connections and how they can inform development of CBTp treatment models. Given the economic and practical barriers that limit the use of neuroimaging in routine clinical practice, it will also be important to identify pragmatic behavioural analogues for these brain connectivity markers of social affective processing. Third, although the inclusion of a clinical control group allowed greater confidence in attributing the changes in CBTp receivers to the intervention, we cannot rule out the possibility that the changes reflect symptom improvement that are non-specific to CBTp, because symptomatic improvement differed between the groups. However, this issue is mitigated by the finding that only two of the 18 connectivity changes previously correlated with pre- and post-CBTp symptom improvement.^14^ Studies contrasting CBTp and pharmacotherapy are ultimately warranted to examine the specificity of these psychobiological processes. A related point is that the medication that CBTp completers received is likely to also have formed an important part of their overall recovery. Finally, because allocation to CBTp (vs treatment-as-usual) was not randomised, we cannot rule out the possibility of a selection bias. There were no explicit biases in recruitment and the CBTp group did not show any differences in any of the clinical, demographic or neural measures included in the study.^16^ However, we previously reported that verbal intelligence (but not in other cognitive functions) was elevated in the CBTp completers compared with treatment-as-usual participants.^43^ It is possible that this conferred an advantage in terms of response to CBTp.

Our previous investigation^14^ provided evidence that CBTp leads to substantial reorganisation of functional connectivity supporting social affective processing, relatively little of which is captured by measures of symptom change. The present findings extend this work by providing initial evidence that it is the degree to which this reorganisation takes place that determines sustained gains in the long-term recovery of people with psychosis. This justifies further work utilising this novel methodology on a larger scale.

## Figures and Tables

**Table 1:** Demographics, task performance and clinical characteristics of participants

	*Entire CBTp group (*n=*22, 18 male)*		*Long-term follow-up (*n=*15, 11 male)*		*Group difference*
	*Mean (s.d.)*		*Mean (s.d.)*		
Age (years)	35.7 (7.82)		37.9 (7.56)		*t*(20)=2.14, *P*=0.045
Education (years)	13.9 (3.26)		14.1 (3.08)		*t*(20)=0.324, *P*=0.75
Predicted IQ^a^	109.4 (9.68)		110.4 (8.14)		*t*(20)=0.68, *P*=0.5
Age at illness onset	24.8 (8.38)		26.4 (8.99)		*t*(20)=1.48, *P*=0.15
Duration of illness (years)	10.9 (7.70)		11.4 (8.76)		*t*(20)=0.429, *P*=0.67
Medication	Atypical antipsychotic (*n*=20); combined atypical and typical (*n*=2)		Atypical antipsychotic (*n*=14); combined atypical and typical (*n*=1)		
Chlorpromazine equivalent (mg)	543 (479.3)		512.9 (450)		*t*(20)=0.295, *P*=0.77
					
	*Pre-therapy*	*Post-therapy*	*Pre-therapy*	*Post-therapy*	
*Gender discrimination accuracy (%)*					
Neutral	92.6 (10.8)	91.8 (13.1)	91 (14.1)	90.8 (10.5)	F(1, 20)=0.122, *P*=0.73
Fear	90.5 (14.4)	91.4 (16.5)	92.1 (11.2)	91.7 (15.7)	F(1, 20)=0.267, *P*=0.61
Anger	88.6 (15.2)	88.9 (14.2)	94.8 (9.6)	94.2 (7.6)	F(1, 20)=0.045, *P*=0.84
Happy	94.7 (8.48)	93.3 (9.94)	94.2 (10.4)	94 (8.6)	F(1, 20)=0.331, *P*=0.57
					
*Detection (%)*					
* *No face	93.4 (12.4)	91.5 (16.4)	92.4 (13.2)	92.7 (9.5)	F(1, 20)=0, *P*=0.98
					
*PANSS*^b^					
Positive symptoms	18.1 (4.84)	14.9 (4.10)^c^	17.7 (4.4)	14.3 (4)^d^	F(1, 20)=0.089, *P*=0.77
Negative symptoms	17.7 (4.23)	15.6 (4.29)^c^	17.3 (4.4)	15.6 (4.4)^d^	F(1, 20)=0.7, *P*=0.41
General psychopathology	33.5 (7.24)	28.6 (7.40)^c^	32.6 (5.6)	27.2 (6.8)^d^	F(1, 20)=0.22, *P*=0.64
Total symptoms	69.3 (13.3)	59.0 (14.7)^c^	67.5 (11.1)	57.1 (14)^d^	F(1, 20)=0.02, *P*=0.9
					
Beck Depression Inventory	16.2 (8.3)^e^	11.5 (9.9)c,^e^	16.7 (9.7)	9.9 (10.2)^d^	F(1, 18)=2.34, *P*=.11
Rosenberg self-esteem	24.8 (6.3)	22.7 (5.3)	24 (6.3)	22.6 (5.3)^d^	F(1, 20)=0.74, *P*=0.4
					
*Beck cognitive insight scale*					
Self-certainty	5.5 (3.5)	4.1 (4)	5.2 (3.6)	4.7 (4.6)	F(1, 20)=1.23, *P*=0.27
Self-reflectiveness	17.3 (5.8)	14.9 (5.7)	17.3 (5.9)	15.3 (5.7)	F(1, 20)=0.01, *P*=0.93
Composite	11.8 (6.9)	10.9 (7.3)	12.1 (7)	10.8 (7.5)	F(1, 20)=0.75, *P*=0.4
					
Birchwood insight scale	10.1 (2.1)	9.9 (2)	10.2 (1.6)	10.1 (1.4)	F(1, 20)=0.18, *P*=0.68

Abbreviations: CBTp, cognitive behavioural therapy for psychosis; PANSS, Positive and Negative Syndrome Schedule.

a National adult reading test.^25^

b Positive and negative syndrome scale.^26^

c Significant symptom reduction following CBTp previously reported in the full sample.^14^

d We did not test for symptom reductions within the subgroup that was followed up as no group differences in symptom change were found between this subgroup and the full sample (final column).

e Missing data for one participant.

There were no differences in pre- to post-therapy change between the full group previously reported and those available for the present follow-up study in terms of performance, symptom change or other clinical measures.
