# Brain connectivity changes occurring following cognitive behavioural therapy for psychosis predict long-term recovery

**DOI:** 10.1038/tp.2017.194

**Published:** 2017-08-15

**Authors:** L Mason, E Peters, S C Williams, V Kumari

**Addendum to**: *Translational Psychiatry* (2017) **7**, e1001; doi:10.1038/tp.2016.263; published online 17 January 2017

After publication, the authors determined that regression tests are the appropriate *post hoc* tests for clarifying the direction of the three MANOVA results that found significant associations between functional connectivity changes and long-term clinical outcomes. In each of the three regression tests, greater increases in functional connectivity were associated with improved long-term clinical outcomes, as ascertained by positive beta weights for the following regressors: longitudinal positive psychotic symptoms (*β*=0.157, *t*(14)=2.79; *P*=0.015), longitudinal affective symptoms (*β*=0.187, *t*(14)=2.36; *P*=0.035) and subjective long-term recovery (*β*=0.13, *t*(14)=2.56; *P*=0.024). These tests replace the correlation tests originally reported in the article.

