# Brain connectivity changes occurring following cognitive behavioural therapy for psychosis predict long-term recovery

**DOI:** 10.1038/tp.2017.195

**Published:** 2017-08-15

**Authors:** L Mason, E Peters, S C Williams, V Kumari

**Correction to:** *Translational Psychiatry* (2017) **7**, e1001; doi:10.1038/tp.2016.263; published online 17 January 2017

Throughout the text, the tests were incorrectly described as being one-tailed. The text should have stated that all tests were two-tailed.

