# Supplementary material for: Brain connectivity changes occurring following cognitive behavioural therapy for psychosis predict long-term recovery
Source: Transl Psychiatry. 2017 Jan 17;7(1):e1001–. doi: 10.1038/tp.2016.263 (PMC5545728; doi:10.1038/tp.2016.263)
Supplement: Supplementary Information [file tp2016263x1.docx]

### **Supplementary Materials:**

Clinician ratings procedure and inter-rater reliability

For all variables, we rated ‘unknown’ if information could not be determined from case note entries for that month or inferred retrospectively from other months. For a small number of participants (n = 2) who were discharged from the care of the mental health trust (either to primary care with their general practitioner and not re-referred, or because of relocating to another area), ratings were made up until point of discharge, provided there were at least three years of ratings.

### Ratings were performed by the first author (LM; R1). To establish reliability, a randomly selected subsample (n = 8; 54%) were additionally rated by an independent clinical psychologist (R2). Agreement between the two raters was assessed using interclass correlations within a one-way random model [^49^](#_ENREF_49). The degree of inter-rater agreement was interpreted from these coefficients according to standard criteria [e.g. ^50^](#_ENREF_50). Inter-class correlations (ICCs) indicated that inter-rater agreement was “almost perfect” for months in non-remission [ICC(1) = .86; Mean _R1_ = 11.0%, SD _R1_ = 14.6, Mean _R2_ = 6.39%, SD _R2_ = 11.8], “moderate” for partial-remission [ICC(1) = .8; Mean _R1_ = 83.6%, SD _R1_ = 23.5; Mean _R2_ = 78.8%, SD _R2_ =22.8] and “moderate” for full-remission [ICC(1) = .38; Mean _R1_ = 5.22%, SD _R1_ = 10.9; Mean _R2_ = 14.2%, SD _R2_ = 15.7]. For affective symptoms status, agreement for months with low affective symptoms was “almost perfect” [ICC(1) = .89; Mean _R1_ = 87.5%, SD _R1_ = 10.9, Mean _R2_ = 85.8%, SD _R2_ = 17.0], “moderate” for moderate affective symptoms [ICC(1) = .61; Mean _R1_ = 10.9%, SD _R1_ = 9.61, Mean _R2_ = 10.0%, SD _R2_ = 13.5] and “moderate-to-moderate” for high affective symptoms [ICC(1) = .47; Mean _R1_ = 1.86%, SD _R1_ = 2.95, Mean _R2_ = 2.75%, SD _R2_ = 5.34].

### Use of longitudinal symptom ratings

### For each longitudinal symptom domain, we sought to preserve all explanatory variables rather than to collapse them together. The raw variables were highly correlated (Supplementary Table 1) however, which led to unacceptable levels of collinearity: variance inflation factor (VIF) = 11.8, exceeding recommended thresholds of VIF less than 4-10 [^51-53^](#_ENREF_51). To avoid unacceptable collinearity between these raw variables, we calculated difference scores between the low and high symptom categories: for psychotic symptoms (percentage of months in non-remission subtracted from percentage of months in full remission) and affective symptoms (percentage of months with severe symptoms subtracted from percentage of months with low symptoms). These difference variables yielded acceptable levels of collinearity when entered into the main multivariate analysis of variance (MANOVA; VIF ≤ 3.57). Next we reduced the longitudinal measures to a single variable, per symptom domain, that could be used as a predicted variable for the clinical and functional connectivity predictors. To this end we computed the residual from the variables used in the main analysis, separately for the psychotic and affective symptom domains. For positive psychotic symptoms we regressed percentage of months in partial remission onto the difference between full- and non- remission, whereas for affective symptoms we regressed percentage of months with “moderate” affective symptoms onto the difference between “low” and “severe” affective symptoms.

Additional clinical measures

### Clinical and cognitive measures of insight were assessed pre- and post- CBTp, using the Birchwood Insight Scale [^29^](#_ENREF_29) and Beck Cognitive Insight Scale [^30^](#_ENREF_30), respectively. Self-esteem was also available for these time points, as assessed using the Rosenberg Self-Esteem Scale [^31^](#_ENREF_31).

### At long-term follow-up we acquired the Warwick-Edinburgh Mental Well-being Scale [WEMWBS, ^34^](#_ENREF_34), CHoice of Outcome in CBT for Psychotic Experiences [CHOICE, client version, ^35^](#_ENREF_35) and the Work and Social Adjustment Scale [^36^](#_ENREF_36). These were not included in analyses due to missing observations and high correlation with our primary long-term outcome; subjective recovery.

### Image acquisition and functional connectivity analysis

240 T2*-weighted images were acquired using a 1.5 Tesla General Electric Signa system with the following parameters: echo time 40 ms, repetition time 3 s, flip angle 90^0^, field of view 240 mm, slice thickness 7.0 mm, interslice gap 0.7 mm. In the same session, a high-resolution structural scan (T1-weighted images in the axial plane with 1.5 mm contiguous sections) was acquired using a 3D inversion recovery prepared spoiled gradient recalled acquisition (echo time 5.1 ms, repetition time 18 ms, inversion time 450 ms, flip angle 20 degrees with one data average, 256x256x128 voxel matrix).

Symptom specificity of predictions from functional connectivity data

To test the unique contribution of the two longitudinal measures directly, we performed a further analysis in which both symptom residuals were entered as regressors into a common model, with the connectivity changes as dependent variables (see Supplementary Table 3). Degree of CBTp-led change in amygdala connectivity with IPL remained a significant predictor of affective symptoms (p = .001) and the change in DLPFC connectivity with somatosensory cortex also remained a marginally significant predictor of positive psychotic symptoms (p = .066). A new finding was that the amygdalo-IPL connection which predicted affective symptoms (in this analysis and previously), was also a predictor of positive psychotic symptoms (p = .02). This suggests that affective and positive psychotic symptoms have somewhat overlapping psychobiology, with a common amygdalo-IPL connection predicting both symptom domains.

Comparing functional connectivity markers against symptoms measures for predicting long-term outcomes

We used stepwise regression analysis to test the relative contributions of the functional connectivity and symptom measures (T1 to T2) in predicting long-term outcomes (T2 to T3).

We first tested which of the clinical measures, if any, predicted long-term outcomes. To this end we repeated the main MANOVAs with the clinical measures entered as the dependent variables in place of the connectivity changes (and the longitudinal psychotic and affective symptoms remaining as regressors in separate MANOVAs). Because our aim was to populate a list of candidate variables that might outperform the functional connectivity approaches, we took an inclusive approach without correcting for these multiple tests. We first examined the primary symptom measures by entering change (between T1 and T2) in psychotic (from the Positive and Negative Symptoms Schedule; PANSS) and depressive (Beck Depression Inventory; BDI) symptoms. We also checked whether absolute symptom levels were predictors by including pre– (T1) and post- (T2) therapy scores on the PANSS and BDI. Next we tested insight (both clinical and cognitive forms) and self-esteem as predictors. As there had been no significant change on these measure following CBTp [^38^](#_ENREF_38), only the pre-therapy (T1) scores were entered into the MANOVA.

There were only effects for clinical insight, which was significant for affective symptoms [F(1, 13) = 8.22, p = .013] and approached significance for psychosis symptoms [F(1, 13) = 3.47, p = .085]. There were no effects for psychotic symptoms (PANSS: positive, negative and general psychopathology subscales) nor in depression (BDI) (all p’s ≥ .23) level, cognitive insight (p ≥.46), or self-esteem (p ≥ .22).

Using stepwise regression analysis, we first tested the predictive value of clinical insight for positive psychotic symptoms and then for affective symptoms, separately. In both models, clinical insight was entered as a predictor in a first step. In the second step, the significant connection was entered for the respective symptom domain: DLPFC-postcentral gyrus (for positive psychotic symptoms) and Amygdalo-IPL (for affective symptoms). We tested whether this could account for significant additional variance compared to the first step.

For positive psychotic symptoms, the first-step model was at trend level (p = .085) and clinical insight explained 21.1% of the variance. At second step the model became significant [F(2, 12) = 5.1, p = .025; F change also significant: F(1, 12) = 5.51, p = .037] and change in DLPFC-postcentral gyrus connectivity explained a significant amount of additional variance (a further 23.5%; standardised b = .522, t = 2.35, p = .037).

For affective symptoms, the first-step model was significant [F(1, 13) = 8.22, p = .013], with clinical insight explaining 34% of the variance. The model remained significant when adding amygdalo-IPL connectivity change [F(2, 12) = 7.76, p = .007] and the increase in F value was also significant: F(1, 12) = 4.86, p = .048]. The connectivity regressor was significant (standardised b = .448, t = 2.2, p = .048) and explained an additional 17.7% of the variance above clinical insight alone.

Validating main analysis using raw longitudinal symptom variables

### To corroborate the main analysis we ran a MANOVA substituting the residualised symptom variables with the raw data variables. In this way the difference scores were entered as regressors in two separate MANOVA along with the remaining variable for the relevant symptom domain (i.e. percentage of months in partial remission and percentage of months with moderate affective symptoms for the psychosis and affective symptom MANOVA, respectively). We performed this on all connections that had previously shown significant Group-by-Time interactive effects. The same connections were identified as important using this approach as in the main analysis (Supplementary Table 4).

Supplementary Table 1. Average monthly ratings from clinician entries in patient records for symptoms, level of care and occupational functioning.

| *% months* | | *Mean (SD)* |
| --- | --- | --- |
| *Psychotic symptoms* | Non-remission | 6.5 (11.7) |
|  | Partial remission | 65.8 (39.8) |
|  | Full remission | 27.2 (39.8) |
|  |  |  |
| *Affective symptoms* | Low | 88.2 (15.1) |
|  | Moderate | 10.5 (14.8) |
|  | Severe | 1 (2.3) |
|  |  |  |
| *Level of Care* | Discharged from services | 7.1 (17.7) |
|  | Outpatient | 87.7 (20.4) |
|  | Home treatment | 1.6 (3.8) |
|  | Inpatient care | 3.1 (8.8) |
|  |  |  |
| *Occupational functioning* | Employed | 28.2 (44.7) |
|  | Volunteering/training | 20 (34.4) |
|  | Unemployed | 50.3 (44.1) |

Supplementary Table 2. Correlations between longitudinal case note measures. Values are Pearson correlation coefficients with significance value in brackets.

| Variable (% months) | | % months | | |
| --- | --- | --- | --- | --- |
|  |  | Non-remission | Partial-remission | Full-remission |
| Positive psychotic symptoms | Non-remission |  | -0.08 (0.78) | -0.21 (0.45) |
|  | Partial remission | -0.08 (.78) |  | -0.96 (<.001) |
|  | Full remission | -0.21 (.45) | -0.96 (<.001) |  |
|  |  |  |  |  |
| Affective symptoms | Low | -0.40 (.14) | 0.30 (0.29) | -0.18 (0.52) |
|  | Moderate | 0.23 (.32) | -0.21 (0.46) | 0.14 (0.62) |
|  | Severe | .91 (<.001) | -0.14 (0.62) | -0.13 (0.64) |
|  |  |  |  |  |
| Level of Care | Discharged | -0.17 (.54) | -0.34 (0.21) | 0.39 (0.16) |
|  | Outpatient | -0.37 (.18) | 0.47 (0.08) | -0.35 (0.21) |
|  | Home Treatment | .88 (<.001) | -0.31 (0.26) | 0.05 (0.86) |
|  | Hospitalised | .85 (<.001) | -0.22 (0.43) | -0.03 (0.9) |
|  |  |  |  |  |
| Occupational functioning | Employed | -0.30 (.28) | -0.20 (0.47) | 0.3 (0.28) |
|  | Voluntary/Course | 0.16 (.58) | 0.28 (0.31) | -0.33 (0.23) |
|  | Unemployed | 0.19 (.50) | 0.01 (0.98) | -0.07 (0.81) |

Supplementary Table 3. Univariate effects for both longitudinal measures when entered as regressors into a common multivariate analysis of variance model. Each pair of measures was separately reduced to its residual (see results). Significant effects are in bold. Abbreviations: amygdala (AMYG), dorsolateral prefrontal cortex (DLPFC), inferior parietal lobule (IPL).

| Condition | Connection | Psychotic Symptoms | Affective Symptoms |
| --- | --- | --- | --- |
| Social threat | AMYG-/DLPFC | F(1,12) = 1.92 , p = 0.19 | F(1,12) = 1.21 , p = 0.29 |
|  | AMYG-Occipitotemporal area/IPL | **F(1,12) = 7.18 , p = 0.02** | **F(1,12) = 17.7, p = .001** |
| Prosocial | DLPFC-Postcentral gyrus | **F(1,12) = 4.08 , p = 0.07** | F(1,12) = 0.16 , p = 0.70 |

Supplementary Table 4. Univariate effects of longitudinal positive psychotic symptoms and affective symptoms on change in functional connectivity from amygdala (AMYG) and dorsolateral prefrontal cortex (DLPFC). Monthly ratings were made of psychotic symptoms (whether in full, partial or non-remission) and affective symptoms (whether absent/low, moderate or severe). The percentage of months in full remission minus non remission and percentage of months in partial remission were entered as regressors into multivariate analysis of variance, as were the difference in percentage of months (severe over low) and percentage of months with moderate affective symptoms (see Methods). Significant effects are in bold. Abbreviations: inferior parietal lobule (IPL); superior parietal lobule (SPL); subgenual anterior cingulate cortex (sgACC), dorsal anterior cingulate cortex (dACC),

| Condition | Connection | Difference in psychotic symptoms (full remission over non remission) | Psychotic symptoms (partial remission) | Difference in affective symptoms (severe over low) | Affective Symptoms (moderate severity) |
| --- | --- | --- | --- | --- | --- |
| *Angry* | AMYG-Posterior cingulate | F(1, 12) = 0.79 , p = 0.39 | F(1, 12) = 0.06 , p = 0.82 | F(1, 12) = 0.49 , p = 0.50 | F(1, 12) = 0.24 , p = 0.64 |
|  | AMYG-DLPFC | F(1, 12) = 0.68 , p = 0.43 | F(1, 12) = 0.07 , p = 0.80 | F(1, 12) = 0.00 , p = 0.97 | F(1, 12) = 0.02 , p = 0.89 |
|  | AMYG-SPL | F(1, 12) = 0.31 , p = 0.59 | F(1, 12) = 0.12 , p = 0.73 | F(1, 12) = 3.58 , p = 0.08 | **F(1, 12) = 5.11 , p = 0.04** |
|  | AMYG-Motor cortex | F(1, 12) = 0.59 , p = 0.46 | F(1, 12) = 1.42 , p = 0.26 | F(1, 12) = 2.77 , p = 0.12 | F(1, 12) = 3.50 , p = 0.09 |
|  | AMYG- IPL | F(1, 12) = 0.40 , p = 0.54 | F(1, 12) = 1.82 , p = 0.20 | **F(1, 12) = 7.27 , p = 0.02** | **F(1, 12) = 7.34 , p = 0.02** |
|  | AMYG-Thalamus | F(1, 12) = 0.01 , p = 0.94 | F(1, 12) = 0.00 , p = 1.00 | F(1, 12) = 0.00 , p = 0.99 | F(1, 12) = 0.06 , p = 0.82 |
|  | DLPFC-sgACC | F(1, 12) = 0.24 , p = 0.64 | F(1, 12) = 0.04 , p = 0.84 | F(1, 12) = 1.28 , p = 0.28 | F(1, 12) = 1.98 , p = 0.19 |
|  | DLPFC-dACC | F(1, 12) = 0.14 , p = 0.72 | F(1, 12) = 0.91 , p = 0.36 | F(1, 12) = 2.95 , p = 0.11 | F(1, 12) = 3.71 , p = 0.08 |
|  | DLPFC-Posterior cingulate / IPL | F(1, 12) = 0.06 , p = 0.82 | F(1, 12) = 0.31 , p = 0.59 | F(1, 12) = 0.00 , p = 0.97 | F(1, 12) = 0.00 , p = 0.98 |
|  |  |  |  |  |  |
| *Happy* | DLPFC-Thalamus | F(1, 12) = 2.04 , p = 0.18 | F(1, 12) = 0.97 , p = 0.34 | F(1, 12) = 0.20 , p = 0.67 | F(1, 12) = 0.36 , p = 0.56 |
|  | DLPFC-Postcentral gyrus | **F(1, 12) = 9.13 , p = 0.01** | F(1, 12) = 2.63 , p = 0.13 | F(1, 12) = 1.54 , p = 0.24 | F(1, 12) = 2.75 , p = 0.12 |
|  | DLPFC-Superior Temporal Gyrus | F(1, 12) = 0.03 , p = 0.86 | F(1, 12) = 0.20 , p = 0.67 | F(1, 12) = 0.27 , p = 0.61 | F(1, 12) = 0.22 , p = 0.65 |
